# How Work Stress Impacts Emotional Outcomes of Chinese College Teachers: The Moderated Mediating Effect of Stress Mindset and Resilience

**DOI:** 10.3390/ijerph191710932

**Published:** 2022-09-01

**Authors:** Tao Yu, Jiayuan Li, Lidong He, Xiaofu Pan

**Affiliations:** 1 College of State Governance, Southwest University, Chongqing 400715, China; 2 Department of Human Resources, Southwest University, Chongqing 400715, China; 3 College of International Education, Sichuan International Studies University, Chongqing 400031, China

**Keywords:** work stress, stress mindset, resilience, emotional exhaustion, positive affect, negative affect, Chinese college teachers

## Abstract

Based on the job demands-resources model and conservation of resource theory, this study investigated 456 Chinese college teachers’ work stress, stress mindset, resilience, emotional exhaustion, positive affect, and negative affect. The results of mediation analysis showed that resilience played a partial mediation role between work stress and emotional outcomes (emotional exhaustion, positive affect, and negative affect). Moreover, the results of a moderated mediation analysis showed that stress mindset moderated the relationship between work stress and resilience, and moderated the mediating effect of resilience between work stress and emotional outcomes (emotional exhaustion, positive affect, and negative affect). Specifically, work stress had a significant negative predictive effect on resilience when stress mindset is low (*β* = −0.54, *p* < 0.001); work stress could also negatively predict resilience when the stress mindset is high (*β* = −0.47, *p* < 0.001), but its effect decreased, and stress mindset negatively moderated the path between work stress and resilience. Finally, we discussed theoretical implications, practical implications, limitations, and future directions.

## 1. Introduction

With the development of science, technology, and increasingly fierce competition, high work stress has become a common phenomenon among employees in all walks of life nowadays. In the field of higher education, the work stress of college teachers is increasing. A significant harm of college teachers’ work stress is that it will damage their physical and mental health, including the occurrence of more abnormal physiological indicators such as hypertension, hyperglycemia, thyroid nodules and breast nodules [1], as well as the damage to job satisfaction and job burnout [2]. Considering the relationship between work stress and emotion, this study regards emotional exhaustion, positive affect, and negative affect as the main consequence of college teachers’ work stress.

Although previous studies have explored the direct impact of work stress on the emotional outcomes of college teachers [3], it should be noted that the impact of work stress on emotional outcomes is not always direct, but may also be affected by the indirect effect of some factors. This study focuses on the indirect impact of resilience as an mediating role on the emotional outcomes, as previous studies had found that work stress had a negative impact on resilience and burnout [4].

However, work stress is not always harmful. According to the literature on a stress mindset, how individuals view stress is more important than how much stress they perceived [5]. Those who believe that stress is harmful will suffer from the negative effects traditionally associated with stress. On the contrary, those who do not believe that stress is harmful may buffer the negative effects of stress. If so, college teachers can change their stress mindset to help them cope with work stress effectively. Unfortunately, few researchers have paid attention to the buffering effect of a stress mindset on perceived work stress and emotional outcomes, and the role of a stress mindset in protecting individuals’ resilience from work stress, although these effects are worth studying. Therefore, the purpose of this study is to explore the relationship between work stress, stress mindset, resilience, emotional exhaustion, positive affect, and negative affect, by taking Chinese college teachers as the object.

## 2. Literature Review and Hypotheses Development

### 2.1. Work Stress and Emotional Outcomes of College Teachers

College teachers in all countries around the world generally experience high work stress. The problem of work stress of Chinese college teachers is particularly prominent. For example, a survey found that 44% of Chinese college teachers reported that their work stress was very high [6], and 81% of Chinese college teachers felt that their work brought moderate and severe work stress [7]. This may be caused by various factors. Firstly, with the continuous development of internet technology and artificial intelligence technology, Chinese colleges have put forward more demands for work, so college teachers need to carry out teaching and scientific research through new technical means. According to the job demands-resources model [8], these increasing job demands will bring extra work stress to college teachers. Secondly, with the proposal of China’s educational modernization and strengthening education, college teachers need to shoulder more professional responsibilities and historical missions in addition to completing daily works, and need to devote additional time and energy to teaching and scientific research. Thirdly, with the deepening of the reform of the personnel system in Chinese colleges, many colleges have carried out teacher employment systems and highlighted high-quality performance assessments. This reform of the employment and management system has also increased the work stress on college teachers to stay and promote [1].

Based on the job demands-resources model [8], the demands of work on college teachers’ physical, psychological, and social abilities required their efforts, which will bring high work stress and cause emotional problems. Hence, college teachers’ work stress may lead to less positive affect, more negative affect, and more emotional exhaustion. Positive affect, which is also called positive emotions in some of the literature, refers to a person’s feeling of enthusiasm, activity, alertness, as well as other positive moods and emotions [9]. In contrast, negative affect refers to a person’s feelings of anger, fear, and tension, as well as other painful and unpleasant moods and emotions. Positive affect and negative affect reflect the affective well-being, so they are also often used to measure a person’s subjective well-being together with life satisfaction. Emotional exhaustion, which is the core dimension of job burnout, refers to the emotional resource exhaustion that occurs when individuals in the workplace experience a long-term loss of resources under work and interpersonal stress [10]. Some empirical research showed that college teachers’ work stress was associated with certain emotional outcomes. For example, college teachers with high work stress feel less of an affective well-being. Work stress from scientific research, workload, career development, promotion, and organizational functions would reduce the affective well-being of college teachers [3]. In addition, college teachers’ work stress might also lead to emotional exhaustion [2]. When college teachers are busy coping with work stress, such as student guidance, academic competition, work burden, administrative affairs, international exchanges, etc., they have higher emotional exhaustion [11]. In summary, according to the job demands-resources model [8], when college teachers face higher work demands, they have higher work stress, which will in turn affects emotional outcomes. In other words, when college teachers are under high work stress for a long time, they will experience less positive affect and more negative affect, and are more prone to emotional exhaustion. Therefore, this study puts forward Hypothesis 1: College teachers’ work stress is negatively correlated with their positive affect, and positively correlated with their negative affect and emotional exhaustion.

### 2.2. Mediation of Resilience

The negative effect of work stress on positive affect and the positive effect on negative affect and emotional exhaustion may be mediated by resilience. Resilience refers to the psychological quality that an individual can actively cope with and adapt to when facing dedication or adversity [12]. In the workplace, resilience can also refer to an individual’s positive psychological ability to recover from adversity, uncertainty, conflict, failure, and other situations [13]. In a word, resilience will enable individuals to persevere, quickly recover and grow when they are in adversity or troubled by problems. Therefore, resilience, as an important part of college teachers’ psychological capital, has far-reaching significance for maintaining their emotional function. In fact, a study on college teachers in Mexico had found that the resilience of college teachers could indeed reduce their psychological exhaustion during the COVID-19 pandemic [14]. However, when college teachers are exposed to higher work stress for a long time, their resilience may be decreased. According to the conservation of resource theory [15,16], resilience can be increased or decreased [17]. Living in a rich resource environment will accumulate resource gains, whereas living in a poor resource environment will accumulate resource losses [15]. Continuous work stress will create a poor resource environment for college teachers, which will consume their cognitive resources and lead to the weakening of resilience. Therefore, this study proposes Hypothesis 2: The resilience of college teachers plays a mediating role between their work stress and emotional outcomes (positive affect, negative affect, and emotional exhaustion).

### 2.3. Moderation of Stress Mindset

Although work stress may bring negative consequences to individual resilience and emotional well-being, it may also be affected by individuals’ implicit beliefs about stress, which is called stress mindset [5]. Specifically, Crum et al. (2013) [5] defined two different stress mindsets: one mindset that stress can promote performance and productivity, improve health and happiness, and help with learning and growth is called the stress-is-enhancing mindset; the other one that states that stress will reduce performance and productivity, damage health and happiness, and hinder learning and growth is called the stress-is-debilitating mindset. A stress mindset affects individuals’ stress perception, stress response, and coping style, so it is closely related to individual emotional outcomes [5,18,19]. Compared with individuals with the stress-is-debilitating mindset, individuals with the stress-is-enhancing mindset will generally experience more subjective well-being and less stress, anxiety, and depression [18,20,21]. A stress mindset could regulate the cognitive and emotional results of stress events, in which the stress-is-enhancing mindset could be used as a protective factor to reduce the adverse effects of stress itself. Moreover, the stress mindset will also affect the individuals’ resilience. Those salesmen who hold the stress-is-enhancing mindset had stronger resilience [22]. The stress-is-enhancing mindset enables individuals to construct the positive meaning of perceived work stress. Therefore, even if work stress is high for individuals, it will not damage their resilience when they have a stress-is-enhancing mindset, which in turn maintains their positive affect and protects individuals from emotional exhaustion and negative affect. Therefore, this study proposes Hypothesis 3: College teachers’ stress mindset moderates the mediating effect of resilience between work stress and emotional outcomes (emotional exhaustion, positive affect, and negative affect).

**Figure 1:**
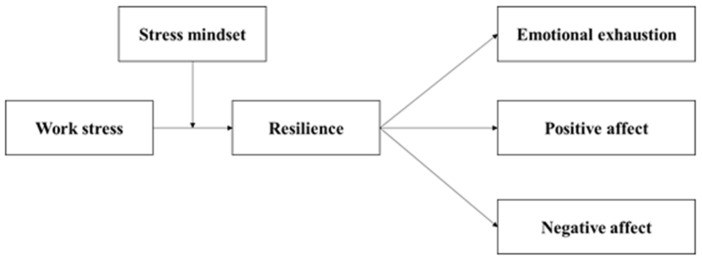
Theoretical model of this study.

The whole theoretical model is as below in Figure 1.

## 3. Materials and Methods

### 3.1. Participants and Procedures

Our data came from a college in Chongqing, China. Through convenient sampling, we distributed paper questionnaires to the teachers of the college. After the teachers filled them out, they were collected by the staff on site. We had distributed 500 questionnaires and effectively recovered 500. After eliminating the invalid questionnaires by the standards of reverse scoring questions and attention screening questions, 456 valid data (91.20%) were finally recovered. Among 456 valid questionnaires, there were 213males (46.71%) and 243 females (53.29%); 389 obtained doctor’s degrees (85.31%), 67 obtained master’s degrees (14.69%); 86 people were under 30 years old (18.86%), 219 people were between 31 and 40 years old (48.03%), 106 people were between 41 and 50 years old (23.25%), and 45 people were over 51 years old (9.87%).

### 3.2. Measurements

In this study, all English scales were translated into Chinese using the “translation-back translation” procedure. The scales were translated into Chinese by two Ph.D. majors in applied psychology, and then checked by a professor of applied psychology and put forward suggestions for revision. Then, an English major graduate student with a psychology background translated the first draft of Chinese into English. Finally, the authors compared the back translation with the original questionnaire and adjusted the Chinese translation according to the differences between the two versions.

#### 3.2.1. Work Stress

To measure college teachers’ work stress, we used the subjective work stress scale of Motowidlo et al. (1986) [23]. Subjective work stress was measured by four items, such as “I feel a great deal of stress because of my job”, and “my job is extremely stressful”. The scale is scored by the 5-point Likert scale, from 1 for “strongly disagree” to 5 for “strongly agree”. Cronbach α of the scale in this study is 0.81.

#### 3.2.2. Stress Mindset

To measure college teachers’ stress mindset, we used the general stress mindset scale (SMM-G) by Crum et al. (2013) [5]. SMM-G consists of eight items, four of which measure the stress-is-enhancing mindset (for example, “experiencing stress facilitates my learning and growth”), and four of which measure the stress-is-debilitating mindset (for example, “experiencing stress inhibits my learning and growth”). The scale is scored by the 5-point Likert scale, from 1 for “strongly disagree” to 5 for “strongly agree”. The scoring method reverses the score for the four items of the stress-is-debilitating mindset, and then take the average score of all eight items. Previous research showed that the scale had good reliability and validity in the context of Chinese culture, which can be used to measure stress mindset [24]. Cronbach α of the scale in this study is 0.90.

#### 3.2.3. Resilience

To measure college teachers’ resilience, we used the resilience subscale in the psychological capital questionnaire of Luthans et al. (2007) [13]. There are six questions on the scale, such as “I will solve the problems I encounter at work anyway”, and “when I encounter setbacks at work, it is difficult for me to cheer up and move on (the question was reverse scoring)”. The scale was scored by the 5-point Likert scale, from 1 for “strongly disagree” to 5 for “strongly agree”. The scoring method reversed the score the three items, and then takes the average score of all six items. Cronbach α of the scale in this study is 0.84.

#### 3.2.4. Emotional Exhaustion

To measure college teachers’ emotional exhaustion, we used the emotional exhaustion subscale of the Maslach Job Inventory (MBI) translated and revised by Li and Shi (2003) [25]. Domestic research supported the applicability of the scale in different fields of workers, and the scale had high reliability and validity. This dimension included five items, and the sample items include “work makes me feel exhausted”. The scale was scored by the 5-point Likert scale, from 1 for “strongly disagree” to 5 for “strongly agree”. Cronbach α of the scale in this study is 0.85.

#### 3.2.5. Positive Affect and Negative Affect

To measure college teachers’ positive affect and negative affect, we used the Positive and Negative Effect Schedule (PANAS) of Watson et al. (1988) [9]. College teachers evaluate and judge the degree and frequency of personal affect according to their actual feelings. The positive affect subscale included 10 items, such as “enthusiastic” and “excited”; the negative affect subscale included 10 items, such as “nervous” and “upset”. The scale was scored by the 5-point Likert scale, from 1 for “not at all” to 5 for “extremely”. Cronbach α of the positive affect subscale in this study is 0.71, and Cronbach α of the negative affect subscale in this study is 0.84.

### 3.3. Statistical Analysis

Analysis was conducted using IBM SPSS 21 for Windows. Cronbach’s α for each scale was calculated to confirm their reliability, and correlation analysis was conducted to identify the correlation between the research variables. Furthermore, to determine the relationship between work stress and emotional outcomes, regression analysis was conducted according to the mediation effect verification procedure and moderating effect verification, and re-verified by bootstrapping using SPSS Process Macro. Statistical significance was determined based on a 5% significance level.

## 4. Results

### 4.1. Common Method Deviation

All the variables in this study were from the self-reports of Chinese college teachers, so there might be a common method deviation problem [26]. Harman single factor test was used to test the common method deviation. The results showed that there were nine factors with characteristic roots greater than one, and the variation explained by the first factor was 34.05%, which did not exceed the recommended value by 40% [26,27]. Therefore, it can be considered that there is no serious common method deviation in this study.

### 4.2. Descriptive Statistics and Correlation Analysis

Means, standard deviations, and correlations among the study’s variables are presented in Table 1. All correlations between variables were statistically significant. Specifically, work stress was positively related to emotional exhaustion (*r* = 0.57) and negative affect (*r* = 0.52), negatively related to stress mindset (*r* = −0.32), resilience (*r* = −0.59), and positive affect (*r* = −0.50); stress mindset was positively related to resilience (*r* = 0.68) and positive affect (*r* = 0.51), negatively related to emotional exhaustion (*r* = −0.53) and negative affect (*r* = −0.53); resilience was positively related to positive affect (*r* = 0.66), negatively related to emotional exhaustion (*r* = −0.74) and negative affect (*r* = −0.66). These results provide preliminary support for hypothesis verification.

### 4.3. Mediating Effect of Resilience

The mediation effect of resilience was tested by using model 4 of SPSS macro program process 2.13 [28]. In step 1, work stress had a significant positive impact on emotional exhaustion (*β* = 0.60, *SE* = 0.04, *p* < 0.001) and negative affect (*β* = 0.41, *SE* = 0.03, *p* < 0.001) had a significant negative impact on positive affect (*β* = −0.34, SE = 0.03, *p* < 0.001) after controlling for age, gender, and degree.

In step 2, work stress and resilience were entered into the equation at the same time. Work stress had a significant negative impact on resilience (*β* = −0.51, *SE* = 0.03, *p* < 0.001), resilience had a significant negative impact on emotional exhaustion (*β* = −0.77, *SE* = 0.05, *p* < 0.001), and the direct effect of work stress on emotional exhaustion was 0.20, *SE* = 0.04, *p* < 0.001. The percentile bootstrap test of deviation correction showed that the indirect effect of work stress on emotional exhaustion through resilience was 0.40, *SE* = 0.04, and the 95% confidence interval (CI) was [0.32, 0.49], indicating that resilience played a partial mediation role between work stress and emotional exhaustion.

Resilience had a significant positive effect on positive affect (*β* = 0.43, *SE* = 0.03, *p* < 0.001), and the direct effect of work stress on positive affect was −0.12, *SE* = 0.03, *p* < 0.001. The percentile bootstrap method test of deviation correction showed that the indirect effect of work stress on positive affect through resilience was −0.22, *SE* = 0.03, and the 95% CI was [−0.28, −0.17], indicating that resilience played a partial mediation role between work stress and positive affect.

Resilience had a significant negative effect on negative affect (*β* = −0.45, *SE* = 0.04, *p* < 0.001), and the direct effect of work stress on negative affect was 0.18, *SE* = 0.03, *p* < 0.001. The percentile bootstrap method test of deviation correction showed that the indirect effect of work stress on negative affect through resilience is 0.23, *SE* = 0.04, and the 95% CI was [0.16, 0.32], indicating that resilience played a partial mediation role between work stress and negative affect.

### 4.4. Moderating Effect of Stress Mindset

To test the moderating effect of stress mindset, it is necessary to estimate the parameters of three regression equations by using model 7 of SPSS process macro [28,29]. Equation 1 estimated the total effect of independent variables on dependent variables, equation 2 estimated the moderating effect of the moderating variable between the independent variable and the mediating variable, equation 3 estimated the effect of mediating variable on dependent variables. Specifically, if the model meets the following three conditions, there is a moderated mediating effect: (1) in equation 1, the total effect of work stress on emotional exhaustion, positive affect and negative affect is significant; (2) in equation 2, the effect of work stress on resilience is significant, and the effect of the interaction term between work stress and stress mindset (work stress × stress mindset) on resilience is significant; (3) In equation 3, resilience has a significant effect on emotional exhaustion, positive affect and negative affect. The results are shown in Table 2, Table 3 and Table 4.

It can be seen from the data in Table 2 that work stress had a significant effect on emotional exhaustion, the interaction term between work stress and stress mindset had a significant effect on resilience, and the effect of resilience on emotional exhaustion was significant. Conditions (1)–(3) were met, indicating the existence of moderated mediating effect of work stress on emotional exhaustion.

It can be seen from the data in Table 3 that work stress had a significant effect on positive affect, the interaction term between work stress and stress mindset had a significant effect on resilience, and the effect of resilience on positive affect was significant. Conditions (1)–(3) were met, indicating the existence of moderated mediating effect of work stress on positive affect.

It can be seen from the data in Table 4 that work stress had a significant effect on negative affect, the interaction term between work stress and stress mindset had a significant effect on resilience, and the effect of resilience on negative affect was significant. Conditions (1)–(3) were met, indicating the existence of moderated mediating effect of work stress on negative affect.

**Figure 2:**
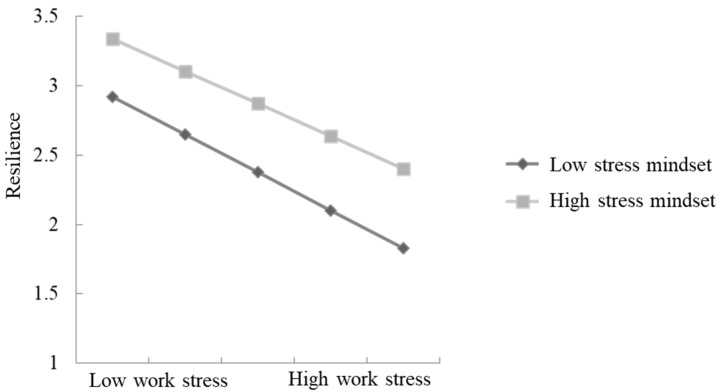
Simple slope analysis of stress mindset.

In order to explain the interaction effect of work stress and stress mindset more clearly, a simple slope analysis was carried out (see Figure 2). It is found that when stress mindset was low, work stress had a significant negative predictive effect on resilience (*β*_simple_ = −0.54, *p* < 0.001); when stress mindset was high, work stress could still negatively predict resilience (*β*_simple_ = −0.47, *p* < 0.001), but its effect decreased. It showed that a stress mindset negatively moderated the path between work stress and resilience.

## 5. Discussion

Given that the work stress of Chinese college teachers is increasing, the mental health and emotional problems of college teachers have become a hot topic in higher education. In view of the potential impact of work stress on emotional outcomes, the purpose of this study is to explore the mediating effect of resilience and the moderating effect of a stress mindset based on the job demands-resources model and conservation of resource theory. Based on the survey data of 456 Chinese college teachers, the present study first examined the direct impact of work stress on emotional outcomes (emotional exhaustion, positive affect, and negative affect). This study then examined the mechanism of the effect of work stress on emotional outcomes (emotional exhaustion, positive affect, and negative affect) through the mediating effect of resilience. Finally, through the examination of the moderating effect of a stress mindset, it is revealed that the influence process of college teachers’ work stress on emotion outcomes (emotional exhaustion, positive affect, and negative affect) depended on the important boundary condition of their own stress mindset. The results provided some theoretical and practical implications for the research on the emotion outcomes of college teachers’ work stress.

### 5.1. Theoretical Implications

Firstly, we found the impacts of college teachers’ work stress on their emotion outcomes, which means this study extended the application of job demands-resources model [8] in the study of college teachers’ perceived work stress. Most of the previous studies on the work stress of college teachers were about the objective evaluation of work stress in specific fields, such as teaching stress and scientific research stress [1,2,3,6,11], ignoring college teachers’ subjective perception of the overall work stress. Different from previous studies, this study focuses on whether college teachers’ subjective perceived overall work stress caused by various demands will affect their emotional experience from the perspective of job demands-resources model, which enriches the existing literature’s understanding of college teachers’ work stress. Specifically, Chinese college teachers’ perceived work pressure will positively impact emotional exhaustion and negative affect, and negatively impact positive affect.

Secondly, we identified the mediating effect of resilience, found the mechanism of negative emotional outcomes of college teachers’ work stress, and expanded the theoretical perspective of college teachers’ work stress research. Most studies on stress and emotion explain the changes of emotion through physiological mechanisms such as autonomic nervous system and hormone changes brought by stress, or explain the emotional response brought by specific stressful life events through emotional event theory. Different from these studies, this study payed attention to the role of resilience in the perception of work stress and emotional outcomes. This is because resilience, as an important psychological resource or psychological capital, will be damaged after encountering high work stress. This result strongly supported the loss spiral hypothesis in the conservation of resource theory [17], that is, when college teachers were in a high work stress situation, their resilience, as a psychological resource, would be further damaged, resulting in negative emotional outcomes. This conclusion not only provides a new theoretical basis for the research of college teachers’ work stress, but also further explains the impact of work stress on emotional outcomes from the perspective of conservation of resource theory, and clarifies its mediating effect.

Thirdly, we explored how to buffer the negative impact of work stress on resilience and emotional outcomes. Most of the previous studies explored the boundary conditions of the effect of work stress from the work characteristics or organizational characteristics, such as work autonomy, organizational support, organizational identity, leadership style, etc. However, Crum et al. (2013) [5] pointed out that individuals’ stress responses were influenced by their interpretation of stress. In the framework of conservation of resource theory [15,16], we examined the moderating effect of stress mindset, made clear the boundary condition for the negative effects of college teachers’ work stress, and made clear that college teachers’ work stress damaged their resilience and emotional outcomes and depended on their stress mindset. The moderating effect of this study on college teachers’ stress mindset showed that college teachers with more stress-is-enhancing mindsets had less loss of resilience when facing higher work stress, so they had more positive emotional outcomes. On the contrary, college teachers with more stress-is-debilitating mindsets had more loss of resilience when facing higher work stress, so they had more negative emotional outcomes. This may be because college teachers with a stress-is-enhancing mindset tend to view work stress as an opportunity to learn and grow, whereas college teachers with a stress-is-debilitating mindset tend to view work stress as a threat to their ability and performance [5]. Therefore, a stress-is-enhancing mindset will protect individuals’ psychological resources from rapid loss; on the contrary, a stress-is-debilitating mindset may accelerate the loss of psychological resources. In conclusion, this study clarified the boundary condition of negative emotional outcomes caused by work stress from the perspective of stress mindset, and expanded the boundary condition on the relationship between work stress and resilience of Chinese college teachers.

### 5.2. Practical Implications

We found that more work stress would make college teachers have lower resilience, lose psychological resources, and then experience less positive affect and more negative affect, as well as emotional exhaustion. Therefore, when human resources departments of colleges arrange the tasks of teaching and scientific research, they need to avoid excessive stress to pursue short-term goals, which will have a long-term adverse impact on the emotional outcomes of college teachers.

In addition, the results showed that work stress had a negative impact on the resilience of college teachers, and then negatively impacted the emotional outcomes. Therefore, in the pre-job training and in-service training of college teachers, colleges should strengthen the intervention and improvement of their resilience, and increase their psychological resources to protect them from producing negative emotional outcomes.

Finally, the results showed that when college teachers held a stress-is-enhancing mindset, the negative effect of work stress on their resilience and emotional outcomes was buffered. Therefore, the human resources department of colleges should consolidate and cultivate the stress-is-enhancing mindset of college teachers and carry out necessary training and group counseling, and psychological intervention to college teachers who have a stress-is-debilitating mindset.

### 5.3. Limitations and Future Directions

This study adopted a self-reported cross-sectional questionnaire, which might have some limitations and could be strengthened in future research. Firstly, although the Harman single factor test was used to examinate the common method deviation, there were still risks. Secondly, this study did not adopt an experimental or longitudinal study design, which makes us limited in explaining the causal relationship between variables. Thirdly, it is impossible to know whether work stress will only cause damage to immediate resilience and emotion outcomes, or will it cause long-term adverse effects. Therefore, future research should further improve the research design. Researchers can adopt the way of teacher-colleague matching or teacher-leader matching, use mixed methods such as questionnaires, experiments, empirical sampling, and other research methods, and add longitudinal research design, such as multi-point questionnaire survey or follow-up of experimental subjects to deepen related researches.

In addition, this study integrates the job demands-resources model and conservation of resource theory, puts forward that college teachers’ stress mindset can increase their psychological resources to meet higher work demands. There may be other theoretical mechanisms. For example, stress mindset is a process of giving meaning to individuals’ stress, which may promote the individuals’ self-regulation process [30,31]. Emotional self-regulation, as an important aspect of self-regulation, may also impact the emotional outcomes of college teachers. When college teachers face high work pressure, stress mindset may also protect their emotional self-regulation ability, or promote their adaptive emotional self-regulation strategies, thus impacting emotional outcomes. Future research can explore the effect of stress mindset from the perspective of self-regulation.

## 6. Conclusions

The data presented in current study suggested that college teachers’ work stress may impact their emotional outcomes. Specifically, work stress negatively impacted positive affect, positively impacted negative affect and emotional exhaustion. Moreover, resilience played a mediating role in the relationship between college teachers’ work stress and emotional exhaustion, in the relationship between college teachers’ work stress and positive affect, and in the relationship between college teachers’ work stress and negative affect. Furthermore, the mediating effect was moderated by their stress mindset. College teachers who viewed stress as debilitating had more resilience, which could help them experience more positive emotional outcomes. Given the functional nature of a stress mindset and resilience, more training and interventions need to be employed to promote college teachers’ emotion well-being.

## Figures and Tables

**Table 1:** Means, standard deviations, and correlations between variables (*N* = 456).

Variables	*M*	*SD*	1	2	3	4	5
1. Work stress	2.92	0.72					
2. Stress mindset	2.35	0.59	−0.32 **				
3. Resilience	2.57	0.64	−0.59 **	0.68 **			
4. Emotional exhaustion	3.51	0.78	0.57 **	−0.53 **	−0.74 **		
5. Positive affect	2.52	0.49	−0.50 **	0.51 **	0.66 **	−0.66 **	
6. Negative affect	3.52	0.57	0.52 **	−0.53 **	−0.64 **	0.63 **	−0.58 **

Note: ** *p* < 0.01.

**Table 2:** Moderated mediating effect of work stress on emotional exhaustion (*N* = 456).

Variables	Equation 1 Emotional Exhaustion			Equation 2 Resilience			Equation 3 Emotional Exhaustion		
	*β*	*SE*	*t*	*β*	*SE*	*t*	*β*	*SE*	*t*
Constant	0.06	0.12	0.55	−0.01	0.07	−0.12	0.00	0.09	0.03
Gender	0.06	0.06	1.03	0.00	0.04	−0.04	0.07	0.05	1.48
Degree	−0.04	0.09	−0.41	−0.03	0.06	−0.50	−0.05	0.07	−0.72
Age	−0.02	0.04	−0.65	0.02	0.02	0.99	0.01	0.03	0.29
Work stress	0.60 ***	0.04	14.07	−0.36 **	0.03	−13.12	0.20 ***	0.04	4.93
Stress mindset				0.60 ***	0.03	17.73			
Int				0.06 *	0.03	2.12			
Resilience							−0.77 ***	0.05	−16.58
*R* ^2^	0.32			0.62			0.58		
*F*	54.17 ***			124.56 ***			124.64 ***		

Notes: Independent variables and dependent variables have been centralized; Int stands for work stress × stress mindset; * *p* < 0.05, ** *p* < 0.01, *** *p* < 0.001.

**Table 3:** Moderated mediating effect of work stress on positive affect (*N* = 456).

Variables	Equation 1 Positive Affect			Equation 2 Resilience			Equation 3 Positive Affect		
	*β*	*SE*	*t*	*β*	*SE*	*t*	*β*	*SE*	*t*
Constant	0.06	0.08	0.74	−0.01	0.07	−0.12	0.09	0.07	1.38
Gender	−0.06	0.04	−1.59	0.00	0.04	−0.04	−0.07	0.03	−2.00
Degree	−0.03	0.06	−0.46	−0.03	0.06	−0.50	−0.02	0.05	−0.38
Age	0.00	0.02	0.14	0.02	0.02	0.99	−0.02	0.02	−0.72
Work stress	−0.34 ***	0.03	−12.02	−0.36 ***	0.03	−13.12	−0.12 ***	0.03	−3.95
Stress mindset				0.60 ***	0.03	17.73			
Int				0.06 *	0.03	2.12			
Resilience							0.43 ***	0.03	12.96
*R* ^2^	0.26			0.62			0.46		
*F*	38.60 ***			124.56 ***			76.91 ***		

Notes: Independent variables and dependent variables have been centralized; Int stands for work stress × stress mindset; * *p* < 0.05, *** *p* < 0.001.

**Table 4:** Moderated mediating effect of work stress on negative affect (*N* = 456).

Variables	Equation 1 Negative Affect			Equation 2 Resilience			Equation 3 Negative Affect		
	*β*	*SE*	*t*	*β*	*SE*	*t*	*β*	*SE*	*t*
Constant	−0.03	0.09	−0.37	−0.01	0.07	−0.12	−0.07	0.08	−0.88
Gender	0.09	0.05	1.92	0.00	0.04	−0.04	0.09	0.04	2.30
Degree	0.00	0.07	0.04	−0.03	0.06	−0.50	−0.01	0.06	−0.10
Age	−0.01	0.03	−0.26	0.02	0.02	0.99	0.01	0.02	0.48
Work stress	0.41 ***	0.03	12.69	−0.36 **	0.03	−13.12	0.18 ***	0.03	5.06
Stress mindset				0.60 ***	0.03	17.73			
Int				0.06 *	0.03	2.12			
Resilience							−0.45 ***	0.04	−11.58
*R* ^2^	0.28			0.62			0.45 ***		
*F*	43.98 ***			124.56 ***			72.36		

Notes: Independent variables and dependent variables have been centralized; Int stands for work stress × stress mindset; * *p* < 0.05, ** *p* < 0.01, *** *p* < 0.001.

## Data Availability

All data supported the findings of this study are available from T.Y. upon reasonable request.
